# Physical Restraint Use in Intensive Care Units: Exploring the Decision-Making Process and New Proposals. A Multimethod Study

**DOI:** 10.3390/ijerph182211826

**Published:** 2021-11-11

**Authors:** María Acevedo-Nuevo, María Teresa González-Gil, María Concepción Martin-Arribas

**Affiliations:** 1 Transplant National Organization, Health Ministry, 28029 Madrid, Spain; 2 Nursing Department, Faculty of Medicine, Autonomous University of Madrid, 28029 Madrid, Spain; mariat.gonzalez@uam.es; 3 Carlos III Health Institute, 28029 Madrid, Spain; comartin@isciii.es

**Keywords:** restraint, physical, critical care, intensive care units, restraint, physical/standards, PAD assessment, multicenter study, multimethod research, mixed-method research

## Abstract

Aim: The general aim of this study was to explore the decision-making process followed by Intensive Care Unit (ICU) health professionals with respect to physical restraint (PR) administration and management, along with the factors that influence it. Method: A qual-quant multimethod design was sequenced in two stages: an initial stage following a qualitative methodology; and second, quantitative with a predominant descriptive approach. The multicenter study was undertaken at 17 ICUs belonging to 11 public hospitals in the Madrid region (Spain) across the period 2015 through 2019. The qualitative stage was performed from an interpretative phenomenological perspective. A total of eight discussion groups (DG) were held, with the participation of 23 nurses, 12 patient care nursing assistants, and seven physicians. Intentional purposive sampling was carried out. DG were tape-recorded and transcribed. A thematic analysis of the latent content was performed. In the quantitative stage, we maintained a 96-h observation period at each ICU. Variables pertaining to general descriptive elements of each ICU, institutional pain-agitation/sedation-delirium (PAD) monitoring policies and elements linked to quality of PR use were recorded. A descriptive analysis was performed, and the relationship between the variables was analyzed. The level of significance was set at *p* ≤ 0.05. Findings: A total of 1070 patients were observed, amounting to a median prevalence of PR use of 19.11% (min: 0%–max: 44.44%). The differences observed between ICUs could be explained by a difference in restraint conceptualization. The various actors involved jointly build up a health care culture and a conceptualization of the terms “safety-risk”, which determine decision-making about the use of restraints at each ICU. These shared meanings are the germ of beliefs, values, and rituals which, in this case, determine the greater or lesser use of restraints. There were different profiles of PR use among the units studied: preventive restraints versus “Zero” restraints. The differences corresponded to aspects such as: systematic use of tools for assessment of PAD; interpretation of patient behavior; the decision-making process, the significance attributed to patient safety and restraints; and the feelings generated by PR use. The restraint–free model requires an approach to safety from a holistic perspective, with the involvement of all team members and the family.

## 1. Introduction

With regard to the Hippocratic maxim, “first do no harm”, nowadays, the need to implement policies that promote safety, quality, excellence, humanized treatment of others, and compliance with ethical aspects in health care settings would seem to be indisputable. In this respect, minimizing the use of physical restraint (PR), and in the event of such use being necessary, judicious application governed by quality standards as well as reducing the risks related with their use, well described in the literature at both a physical and psychosocial level [1,2,3,4,5,6], are seen as a priority in many documents for the control of patient safety issued by agencies such as the Joint Commission on Accreditation of Health care Organizations and the Avedis Donabedian Foundation [7,8,9].

Recent years have witnessed a clear shift on the part of leading consultative/advisory bodies and scientific societies toward a reflexive, judicious, and informed use of restraints, especially since the measure is regarded as being of dubious effectiveness in many cases [8,10,11,12,13]. PR use thus gives rise to a sharp clash between the principles of non-maleficence and beneficence on one hand, and the principle of autonomy on the other [3,11,14,15,16], a clash that is further accentuated by taking into account the adverse effects deriving from their use and viewing the patient from a holistic stance [8]. Turning to aspects of a more regulatory nature, assessment of the appropriateness of PR use should be made in line with generally accepted medical practice (*lex artis*), construed as a concept that is constantly evolving in response to scientific-technical and social advances.

The literature shows a disparity in prevalence figures among countries, partly accounted for by differences in the criteria considered in the respective studies [1,14,17,18,19]. Going by the data issued by the Organization for Economic Co-operation and Development, Spain and other Mediterranean countries rank among the developed countries making greatest use of restraints in all health care settings [8,20].

In the case of critical patients, concern in national and international spheres about the use of PRs is a very recent phenomenon. There are hardly any studies on the topic or reports of positions taken by intensive care units (ICU), and the difference between practice and guidelines seems to be even greater than in other settings; some authors even regard ICU as the “last frontier” of PR use [1,2,17,21,22,23,24,25,26,27,28]. Indeed, it was not until 2010 that one of the first studies on PR use in the critical-patient setting was published, the “Physical Restraint use in Intensive Care units across Europe” (PRICE) study, a pioneering outline of the situation which described the prevalence of 0% to 100% across Europe [1]. Since then, more data on the critical-patient setting have been published: prevalence of PR use stands at around 0% in the United Kingdom and Scandinavian countries; in contrast, 23% of patients admitted to an ICU in Holland had PR, as did 76% of patients with mechanical ventilation in Canada; in Italy, 100% of patients studied had some form of restraint; and while the range fluctuated from 13% to 50% in the USA, it was in the region of 45–50% in Switzerland and France, and 48.4% in South Africa; finally, a prevalence of 15% to 43.9% was reported in Spain [1,3,17,21,23,24,25,27,29,30,31,32]. At all events, regardless of the figures, the wide variability described seems to reflect a degree of complexity in the factors determining use/non-use, which calls for complex in-depth analysis.

In general, health professionals advocate for the need for the use of PRs, seeking justification in individual safety (safety of the patients themselves) or collective safety (safety of third parties), with the latter being almost entirely restricted to mental health [19,33,34]. Individual safety seems to be the principal indication at the ICU, where health professionals almost unanimously cite the prevention of the self-removal of devices [1,15,16,17,35,36,37] as the indication for the use of PRs. However, despite this generalized belief in increased safety—at least physical safety—in light of the evidence, proof of this link has not been found [1,3,17,35,36,37].

### Decision-Making about and Explanatory Models of PR Use

What appears to be evident at the present time is that systematic PR use is becoming increasingly difficult to justify. Despite this, however, the situation persists, and progress must therefore be made in an issue viewed as being highly complex [20,38]. In the search for solutions, initiated in the most advanced fields such as geriatrics and mental health, consideration of the pertinence of restraint measures has highlighted the fact that this is a complex, multifactorial topic, in which a multitude of individual, group, and institutional variables come into play when making decisions about implementing, retaining, or removing the measure [16,20,34,39,40,41].

As noted above, when it comes to making decisions about the placement of PRs, these generally focus on causes that compromise the safety of the patient [33,34,42]. Hence, there are papers that highlight shortfalls in the health professionals’ grasp and management of what the concept “risk versus safety” entails, something that might in turn be linked to the lack of effectiveness of restraints [16,34,39,40,43,44,45,46,47]. The literature points to the existence of other factors such as the organization of clinical units (low nurse-patient ratios, normalized use of restraints without individualized assessment of the need for their use, PR management protocols, etc.); the health professionals themselves (professional experience, skill in finding alternatives to PR, interpretation of patient behavior, etc.); and current legislative aspects that might act as facilitators of PR use and be related to the above-mentioned conceptualizations of risk/safety. However, despite the influence the legislative aspects may have, it is striking that this regulatory framework is unequal at the international level. There are countries such as Spain where the legislative framework is scarce and there is little specifically for critical patients. Thus, the obligatory nature of the medical prescription is hardly indicated, which, on the other hand, is not always fulfilled [20,34,38,39,40,41,48].

Traditionally, nurses have been the main actors in PR administration (essentially, decision-making about placement) and management (placement, retention, and removal) [17,40,46,49,50]. However, there is practically worldwide unanimity to the fact that PR use is a matter of medical prescription in all settings (a requirement that is not met in most countries, with the entire matter of PR administration and management being delegated to professional nursing staff) [8,18,49,51,52]. Given the pivotal role of nurses and the lack of medical prescriptions, most of the studies undertaken to ascertain and comprehend the complexity of the phenomenon have been conducted by nurses with nurses [10,16,50,52,53].

**Figure 1:**
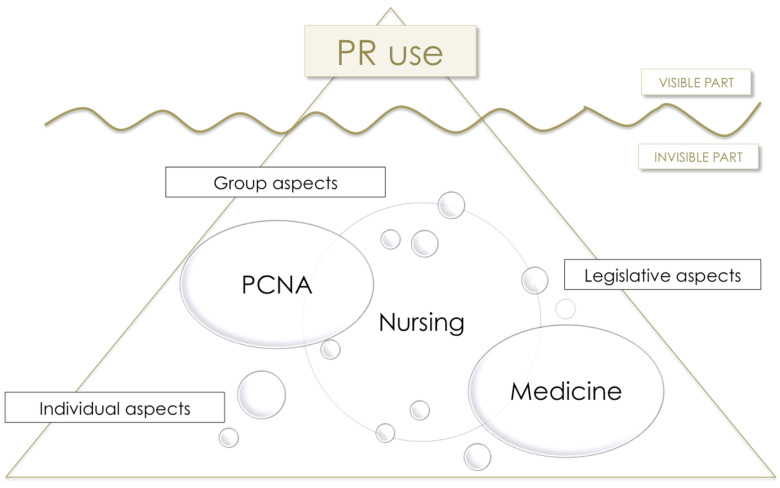
Explanatory model of PR use, “Iceberg Theory”. Abbreviations: PCNA: patient care nursing assistants; PR: physical restraints.

In this respect, although nurses play this central role in PR management, it is reasonable to assume that they are not the only ones involved in decision-making: physicians and patient care nursing assistants (PCNA) could well exert a constant and powerful (and, on many occasions, invisible and contradictory) influence. Acknowledgment of this interdisciplinarity in terms of PR management would be in line with recognition of the complexity of the phenomenon and the existence of individual, group, and legislative factors (as well as the influence of other health care agents) [16,40,41]. It would thus seem reasonable to make use of another of the explanatory theories about PR use in clinical practice, the so-called “Iceberg Theory” [20,38]. According to this theory, the visible part of the iceberg represents PR use, but lower down, an intricate network of interrelated elements is at work; here, all health care actors exert an influence on the main actors in PR use (i.e., the nurses (Figure 1)).

Regardless of who the health professionals tasked with making decisions about PR use might be, the setting in which this might take place, or the elements that might exert an influence, decision-making in clinical practice should include the following five elements: (1) awareness and definition of the problem; (2) determination of the goal; (3) assessment of alternatives; (4) implementation; and (5) evaluation [54]. A number of studies that have sought to approach the decision-making process in the case of PR use in the geriatric and social fields have detected a lack of ability to cite these five, seemingly essential elements, coupled with an inability to discuss each in depth [16,33,34,40,41,45]. According to some authors, this inability to provide a reasoned and informed explanation is an indicator of normalization of PR use together with an absence of reflexive and judicious decisions [16,50]. This notion of a lack of informed decision-making might be linked to the wide variability described in comparable contexts (e.g., between nursing homes or hospitalization units in a given region).

Everything said so far indicates that reflection on the decision-making process for PR use is essential. The inherent complexity of the study of PR, coupled with the difficulties posed by the design of studies that address decision-making, calls for approaches that are reflexive rather than reductionist, and capable of tackling complicated and intricate issues [55].

It should be made clear at this point that, for the purposes of this study, PR was defined as “…any action or procedure that prevents a person’s free body movement to a position of choice and/or normal access to his/her body by the use of any method, attached or adjacent to a person’s body that he/she cannot control or remove easily.” [18]. In line with most of the studies conducted on critical care, full-enclosure side bed rails are excluded from the above definition, in light of the different reasons for their use in ICUs [1,24,27,56].

## 2. Hypothesis and Objectives

The general aim of this study was to explore the decision-making process surrounding PR use and the factors that influence it among the various professionals comprising the ICU health care team (nurses, physicians, and PCNA).

The study hypothesis was formulated under the conceptual proposal of the “Iceberg Theory” [20,38]. Observable differences in PR use in the critical care setting would thus be related to differences in the above-mentioned interlinked and interdisciplinary network of relationships (Figure 1).

Under this conceptual premise, the specific research goals for each of the proposed stages are set out in detail in Table 1 below.

## 3. Methods

**Figure 2:**
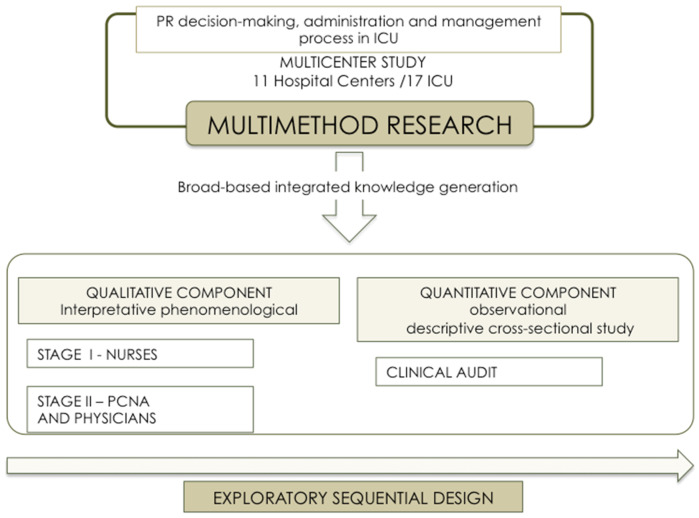
General scheme of the multimethod design. Abbreviations: PCNA: patient care nursing assistants; PR: physical restraint; ICU: intensive/critical care units.

The methodology adopted responds to the need to examine a little explored phenomenon that is also rather complex. Indeed, it was in response to the sheer range of nuanced outcomes envisaged by the specific goals that we chose to adopt a qual-quant multimethod design [57,58,59,60] sequenced in two stages: an initial stage following a qualitative methodology; and a second, quantitative stage in which the predominant approach was descriptive [61]. This sequential dynamic meant that the results obtained via the qualitative study could be used for the design and interpretation of the quantitative component, thereby making for cumulative integration across the research process [62] (see Figure 2).

A multicenter study was thus undertaken at 11 public hospitals in the Madrid Region (Spain) that accounted for a total of 17 ICUs, with the Puerta de Hierro Majadahonda University Teaching Hospital acting as the coordinating hub.

### 3.1. Study Scope

The study covered 17 ICUs belonging to 11 public hospitals in the Madrid region. Although all hospitals participated in the clinical audit, not all did so in qualitative stages I and II, since the respondents were selected on the basis of their suitability and willingness to take part.

Based on the data initially furnished by the hospitals, the nurse:patient ratios of the ICUs involved ranged from 1:2 through to 1:4, according to work shift.

### 3.2. Ethical Considerations

All the health professionals participated in the study voluntarily and were asked to sign the informed consent (IC) form. Confidentiality of information was guaranteed throughout. Once the texts had been transcribed, all the tape recordings were destroyed and the data were anonymized.

ICU data were collected anonymously in the form of aggregate data, with the consent of the directors of the respective ICU being previously obtained for this purpose. The study received the formal approval of all the clinical research ethics committees of the participating hospitals.

### 3.3. Qualitative Interpretative Phenomenological Component

#### 3.3.1. Stage I—Registered Nurses

##### Study Design 

We conducted a qualitative interpretative phenomenological study [4] as proposed by Heidegger [63,64]. The individuals—ICU nurses—were thus deemed to construct different ways of understanding the subject of study (PR use), thanks to social interaction and the development of different ways of perceiving and undergoing the experience of using PR on critical patients, depending on the work setting in which they found themselves (ICU with frequent/systematic, occasional/individualized, and mixed use of PR [64]).

##### Study Scope

Data were collected from December 2013 through January 2015, and generally included health professionals drawn from eight public hospitals and 14 of the ICUs included in the study. Nurse:patient ratios at the ICUs involved ranged from 1:2 to 1:3, according to work shift.

##### Participants and Sampling

We carried out intentional purposive sampling [62,65]. The inclusion criteria were as follows: any nurse who, at the date of study, either was or had been in the ICU for a period of no longer than two years, with a minimum of three years’ clinical experience in ICUs, and who voluntarily wished to participate in the study and agreed to sign the IC form. Considering that nuances and details surrounding PR use could represent an important richness to understand the phenomenon, we decided not to include the professionals who could not remember the use of restraints in critically ill patients recently. We identified professional profiles that might be compatible with different ways of perceiving PR use, according to the clinical setting in question: in each of the discussion groups (DG), we thus sought to mix health professionals drawn from different ICUs who displayed diversity, both in years of professional experience and in the number of different ICUs at which they had worked.

Given the absence of prevalence data at the date of undertaking this stage of the study, the different ICUs were classified by the principal investigator (PI) and research assistants as “systematic or frequent use” (FU), “individualized or occasional use” (OU), or “mixed use” (MU), in line with the frequency and criteria of PR use yielded by a purpose-designed questionnaire (in view of the dearth of validated instruments in the literature) (see Table 2). Access to respondents was obtained through the research assistants [66,67]. Data were collected until theoretical saturation of emerging themes was reached [66,67].

**Figure 3:**
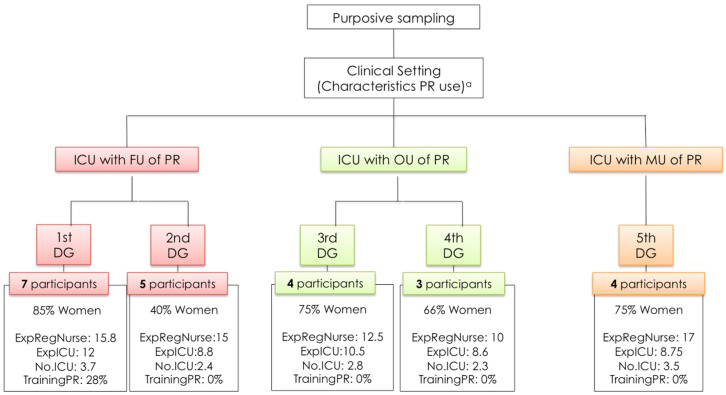
Characteristics of discussion groups according to population criteria and sampling procedures—Registered Nurses. ^a^ Conceptual hypothesis: clinical settings and their organizational cultures influence conceptualizations of PR use. Abbreviations: PR: physical restraint; ICU: intensive/critical care units; FU: frequent/systematic use; OU: occasional/individualized use; MU: mixed use; DG: discussion group; ExpRegNurse: years of work experience as a registered nurse; ExpICU: years of work experience in intensive/critical care units; No.ICU: number of intensive/critical care units other than those where they pursued their professional activity; TrainingPR: percentage of participants with specific training in physical restraints, not necessarily in a critical care setting.

Figure 3 shows the characteristics of the sample and DG, according to the population criteria and sampling procedures used.

##### Data Collection

A total of five DGs were held [67]. In these, based on each respondent’s personal experience of PR management, an effort was made to elicit a shared discourse with respect to the subject of study and the related group and individual factors.

The DGs were tape-recorded and transcribed for subsequent analysis.

All DGs were moderated by the PI, accompanied by some member of the team of qualitative research experts. Initially, use of the same script was envisaged for all the DGs, employing a strategy of nondirective moderation with open and general questions [68], but subsequently new, and more directive questions were introduced to increase discursive productivity.

##### Data Analysis

A thematic analysis of the latent content was performed [69], guided by Colaizzi’s *7*-step method for data-analysis [70]. The procedure followed during this stage was: (1) obtaining a general sense of each transcription; (2) extraction of significant statements; (3) formulation of meanings; (4) sorting of meanings into thematic groups; (5) exhaustive description of the phenomenon of study; (6) description of the fundamental structure of the phenomenon; and (7) validation of findings with participants (member checking) [70]. With the aim of stimulating theoretical sensitivity [71,72], we employed strategies such as the formulation of a glossary of codes, the writing of memoranda, and the use of diagramming techniques [73,74].

Two researchers working in constant liaison took part in the analysis process, mutually agreeing on the individual analysis work completed at each of the above stages. The information generated during each DG was analyzed prior to holding the following session. After analyzing the content of the fifth DG, the topics and subtopics were deemed to be both solid and significant, something that was instrumental in deciding to bring the field work to a close.

Data analysis was performed using the Atlas.ti (version 7.0) computer software package.

##### Rigor Criteria

Epistemological adequacy, adjusted to the subject of study, was monitored in terms of relevance, validity, and reflexivity [75,76]. During the process of analysis, we carried out triangulation among researchers, with sessions for sharing and agreeing upon the inferences made by each of the analysts, member-checking with clinical experts, and some of the respondents [76], and finally, a review of items that should be included in reports of qualitative research quality using the Cosolidated criteria for Reporting Qualitative research (COREQ) checklist [77]. In the case of reflexivity, the proximity of the PI to the study phenomenon may conceivably have entailed an interpretative bias, however, their wide-ranging knowledge and experience of the topic (“being-in-the-world”) [78] were used critically and judiciously, and as a result, contributed favorably to the approach to the research question and analysis. Furthermore, during the undertaking of the study, the PI kept a reflexive diary and made observational, inferential notes/memos, theoretical aids which helped them to identify and express their preconceived ideas [63], and steer the process through the different stages of data collection and analysis.

#### 3.3.2. Stage II—PCNA and Physicians

##### Study Design 

This was a qualitative interpretative phenomenological study [79] that allowed for an in-depth examination of the experiences of PCNA and physicians in terms of PR management in critical patients, and ascertainment of how such experiences might influence nurses, the main actors in the management of PRs in ICUs [22]. In parallel with the previous stage, the fundamental pillars of this design were the group-based construction of the subject of study (PR in ICU) and the approach to this from an empathetic and interpretative standpoint [64,80].

##### Study Scope

Data were collected from December 2015 through March 2017 and included health professionals (PCNA and physicians) from 14 ICUs belonging to 10 secondary- and tertiary-level public hospitals in the Madrid region.

The nurse:patient ratios at all ICUs ranged from 1:2 to 1:3, and the PCNA:patient ratios ranged from 1:4 to 1:5, in both cases depending on the respective work shifts (day versus night). No data on physician:patient ratios were available.

##### Participants and Sampling

We carried out intentional purposive sampling [62,65].

In general, the following inclusion criteria were applied: any professional (physician or PCNA) who, at the date of the DG, was involved in health care activity at the ICU, with a minimum of three years’ clinical experience in an ICU (in the case of PCNA), and who expressly agreed to participate in the study by signing the IC form. In the case of physicians, these were required to have finished their residency training period and be fully qualified as Adjunct Specialists in Intensive Medicine and/or Anesthesiology and Resuscitation.

We considered a series of experience-related criteria that could act as discourse modulators. These included: length of clinical experience in general; length of experience at ICU; and number of ICU at which participants had worked and received specific PR training. Furthermore, other sociodemographic data such as gender and age were likewise recorded.

In addition to the above criteria, and in continuity with the line of work already begun, the ICU to which the health professionals belonged were stratified into “ICU with frequent/systematic use of PR” (ICU with FU of PR) or “ICU with occasional/individualized use of PR” (ICU with OU of PR). In this study, of the total of 14 ICUs covered, eight were ICUs with FU of PR, and six were ICUs with OU of PR.

Access to the respondents was channeled through the research assistants (nurses) who worked at each of the hospitals [66,67]. Data collection made it possible to reach saturation [66,67] when the results were cross-referenced against those previously recorded in the nurses’ DG.

##### Data Collection

A total of three DGs were held, with the participation of 18 health professionals (six per group): two DGs with PCNA (one DG made up of PCNA from ICU with FU of PR, and another made up of PCNA from ICU with OU of PR); and one DG with physicians (mixed group with health professionals from both subtypes of ICU and both specialties).

**Figure 4:**
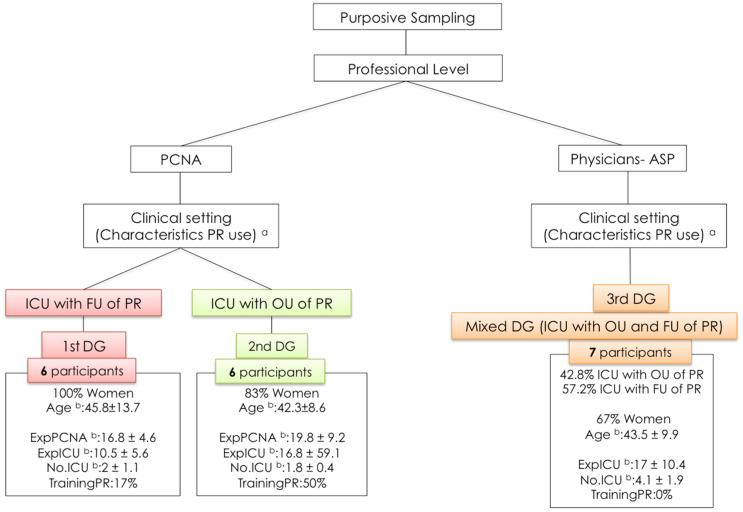
Characteristics of the discussion groups according to population criteria and sampling procedures—PCNA and Physicians. ^a^ Conceptual hypothesis: clinical settings and their organizational cultures influence conceptualizations of PR use. ^b^ Data expressed as mean ± standard deviation. Abbreviations: PCNA: patient care nursing assistants; PR: physical restraints; ExpPCNA: years of work experience as a nursing assistant; ExpICU: years of work experience in intensive/critical care units; ASP: adjunct specialist; TrainingPR: percentage of participants with specific training in physical restraints, not necessarily in a critical care setting; DG: discussion group; No.ICU: number of intensive/critical care units other than those where they pursued their professional activity; ICU: intensive/critical care units; OU: occasional/individualized use; FU: frequent/systematic use.

Figure 4 shows the characteristics of the sample and DG, according to the population criteria and sampling procedures used.

All three DGs were moderated and led by the PI, accompanied by some members of the research team who had experience in qualitative research and acted as an observer during the session. The same script was used for the DG with PCNA.

For the physicians’ DG, after the preliminary analysis of the PCNA’s DG and triangulation with the results of the nurses’ DG from previous stages of the study, a new more directive script was drawn up and geared to specific aspects in order to increase the DG’s productivity.

The DG lasted approximately 90 min and were conducted in a calm atmosphere without interruptions. The sessions were tape-recorded and transcribed by an external, specialized company for subsequent analysis.

##### Data Analysis

We performed a thematic analysis of the free-discourse content on theoretical reference frameworks [81,82]. The results of this analysis were then shared among the qualitative analysts, and discussed, nuanced, and refined to ensure solid, critical integration.

The steps taken to co-construct the meaning of this experience are described below. We analyzed the discourses generated, first by the PCNA, and second by the physicians, in order to then compare common and differential elements, and generate proposals linked to the fact of sharing the selfsame subject of care. To stimulate the capacity for analysis (theoretical sensitivity) [71,72] and integration of meanings, we used diagramming strategies [73,74], which, in their most advanced versions, yielded the figures that illustrate the results.

Despite the fact that, in line with the phenomenological method, analysis of the DG was approached via a thematic analysis, the emergence of meanings called for the use of other strategies such as constant comparison or diagramming, more characteristic of grounded theory. This underscores the nature of qualitative research (i.e., flexible and process-oriented). Furthermore, the sequencing of stages made for a more focused analysis at this second stage, with interpretations of a more explanatory type and constructions that were more conceptual.

The Atlas.ti software package (version 7.5.18) was used for data analysis purposes.

##### Rigor Criteria

We carried out a monitoring process, similar to that performed in the previous stage, with respect to epistemological adequacy in terms of the subject of study and relevance, validity (triangulation of researchers, member checking, and application of the COREQ checklist), and reflexivity (consideration of the PI’s proximity to the subject of study and maintenance of a reflexive diary and memos) [63,70,76,77,78,83].

### 3.4. Quantitative Component—Clinical Audit

Given the sequential nature of the proposed methodology, the design of the clinical audit, rather than being decided at the start, was instead based on the results already obtained in the qualitative stage.

#### 3.4.1. Study Design and Scope

This was an observational multicenter study conducted at 17 ICU belonging to 11 secondary- and tertiary-level public university teaching hospitals from February through May 2016.

#### 3.4.2. Participants and Sampling

The study covered polyvalent ICU, both medical and surgical, and included all patients aged 18 years or over admitted across the study period.

In line with the definition of PR adopted, a patient with restraints was deemed to be any patient to whom some device had been applied at any given time, in order to immobilize him/her or reduce his/her capacity of movement.

#### 3.4.3. Data Collection

The observation period at each ICU was 96 h, a period subdivided into four monthly intervals of 24 h each to enhance the representativeness of the sample.

The research assistant at each ICU recorded the data by direct observation, a review of clinical records (CR), and an interview with the health professionals involved in the care of each patient.

#### 3.4.4. Variables Recorded

We recorded different variables pertaining to the general descriptive elements of each ICU; data on institutional PAD-monitoring policies; and elements linked to the quality of PR use. The set of variables classified in respect of these three criteria can be seen in Table 3. It should be noted here that the variable, “Optimal PR use”, was created by the research team taking into account the variables-elements relating to PR-application quality (Table 3 and Table 4).

**Table 3:** Variables recorded in the clinical audit stage.

Variables Recorded	
General descriptive elements of each ICU	Number of patients admitted; number of patients with PR; number of patients fitted with artificial airways (AAs) (endotracheal tube (ETT), tracheostomy cannula), number of patients with non-invasive mechanical ventilation (NIMV), number of self-removed devices; and type of device. For each ICU, we recorded the nurse:patient ratio, and the existence of a specific written PR protocol and/or specific training of professionals in PR management in critical patients.
Data relating to institutional PAD monitoring policies.	For each ICU, we registered compliance/non-compliance with appropriate PAD monitoring [84,85]. Table 4 shows the definitions for each of these variables.
Elements linked to PR-use quality	In patients with restraints, the following were recorded: concomitant use of PR and analgesia/sedation/neuromuscular blocking agents; location; whether the material applied was certified; time of and indication for use; record of prescription in patient’s CR; existence of a signed consent form, if alternative approaches had been tried prior to use; and adverse physical or behavioral effects related to PR placement [2,84,86]. Based on these variables, a new variable, “Optimal PR use”, was created to give an idea of PR application quality (Table 4 and Table 5).
Elements linked to quantity of PR	In addition to recording overall prevalence and prevalence in patients with AAs or NIMV, a new variable was created, namely, “Compliance with the standard of PR use” (Table 4).

Abbreviations: ICU: intensive/critical care units; AAs: artificial airways; ETT: endotracheal tube; NIMV: non-invasive mechanical ventilation; PR: physical restraint; PAD: pain/agitation-sedation/delirium; CR: clinical records.

**Table 4:** Glossary of variables recorded in the clinical audit stage.

Glossary of Variables [8,14,84,85]	
Specific PR protocol in writing	Existence of a written protocol governing patient management with PR or subsidiary restrictive measures. The protocol must be ICU-specific (general hospital protocol not deemed valid unless it makes specific considerations for ICU).
Specific PR training	Provision of specific training on PR use among critical patients in ICU, open to one or more professional categories.
Appropriate pain monitoring in communicative patients	Institutional regulation requiring a record to be kept of the Numerical Verbal Scale (NVS) or Visual Analogue Scale (VAS) score at least once every nursing shift.
Appropriate pain monitoring in noncommunicative patients	Institutional regulation requiring a record to be kept of the Behavioral Pain Indicator Scale (BPIS), Behavioral Pain Scale (BPS), or Critical Care Observation Tool (CPOT) score at least once every nursing shift.
Appropriate monitoring of sedation	Institutional regulation requiring a record to be kept of the Richmond Agitation Sedation Scale (RASS) or Sedation Agitation Scale (SAS) scores, and/or use of objective systems such as Bispectral Index^®^ (BIS^®^), as the case may be, at least once every nursing shift.
Appropriate monitoring of delirium	Institutional regulation requiring a record to be kept of systematic delirium screening with the Confusion Assessment Method for Diagnosing Delirium in ICU (CAM-ICU) or Intensive Care Delirium Screening Checklist (ICDSC), at least once every 24 h.
Optimal PR use	Based on the bibliographic review, a set of 15 criteria was defined that would reflect optimal PR use. The variable was configured on the basis of 15 criteria, each of which would have a target of 100% compliance (Table 5).
Compliance with the standard of PR use	The ICU were stratified into compliers/non-compliers with the standard, with the compliance cut-off point being set at a use prevalence of 15% or less, the lowest published figure for critical patients in Spain, and an initially desirable goal for all comparable ICU.

Abbreviations: PR: physical restraints; ICU: intensive/critical care units.

**Table 5:** Optimal use of physical restraints—Criteria.

Optimal Physical-Restraint Use—Criteria			
Criteria			Definition Criterion *
Pharmacological management	1	PR use in patients on analgesics	Patients with PR, administered analgesic drugs.
	2	PR use in sedated patients	Patients with PR, administered sedative drugs.
	3	PR use in patients with no NMBAs	Patient with PR, not undergoing treatment with NMBAs (administered in bolus or continuously).
	4	PR use after pharmacological approach ruled out	Patients with PR, after attempt to control symptoms with analgesic and/or sedative drugs.
Non-pharmacological management	5	PR use after verbal or psychological approach ruled out	Patients with PR, after attempt to control symptoms with verbal patient-management skills and invitation to dialogue, while ensuring a calm and soothing atmosphere, and providing information about their process and maintenance of spatio-temporal orientation.
	6	PR use after family approach ruled out	Patients with PR, after attempt to control symptoms, allowing the presence of significant persons/others who would act as a relaxing element for the patient.
	7	PR use after technological-structural approach ruled out	Patients with PR, after suitable adjustment and adaptation of lighting, temperature, video surveillance and proximity to central nursing and patient-monitoring station, etc.
Ethical and legal aspects	8	PR with certified material	Patients with PR applied with certified material, authorized by the institution for this purpose.
	9	PR with written medical prescription	Patients with PR, with written medical prescription in CR for PR and/or type of PR to be used.
	10	PR with written IC	Patients with PR, with authorization via written IC for application of PR.
	11	PR recorded in medical CR	Patients with PR, with written entry in medical CR of PR use.
	12	PR recorded in nursing CR	Patients with PR, with written entry in nursing CR of PR use.
	13	PR recorded in PCNA’s CR	Patients with PR, with written entry in PCNA’s CR of PR use.
Follow-up	14	Re-assessment of need for PR use during every nursing shift	Patients with routine shift-based assessment by a professional ICU staff member of the need to continue using PR.
	15	Assessment of adverse effects of PR during every nursing shift	Patients with a routine shift-based assessment by a professional ICU staff member of the presence of adverse physical and/or psychological PR-related effects.

* Optimal degree of compliance for each of the criteria deemed to be 100%. Abbreviations: PCNA: patient care nursing assistants; PR: physical restraint; NMBAs: neuromuscular blocking agents; CR: clinical records; IC: informed consent.

#### 3.4.5. Data Analysis

We performed the following: a descriptive analysis of categorical variables using absolute and relative frequencies, and a descriptive analysis of numerical variables using the mean and standard deviation, or the median and 25th (P25) and 75th percentiles (P75), according to compliance with the normality assumption.

The relationship between the variables was analyzed using the U Mann–Whitney test for numerical variables and the Chi-squared or Fisher exact test statistic for categorical variables. The numerical variables were compared using Pearson/Spearman correlation analysis. The level of significance was set at p ≤ 0.05.

The statistical software package used was Stata/IC v.15.1.

#### 3.4.6. Rigor Criteria

Following training of the researchers and a pilot test of the case report form, data were simultaneously collected at all ICUs and analyzed on an aggregate basis, with the participating hospitals, units, patients, and health professionals being anonymized for this purpose.

### 3.5. Integration of Results

**Figure 5:**
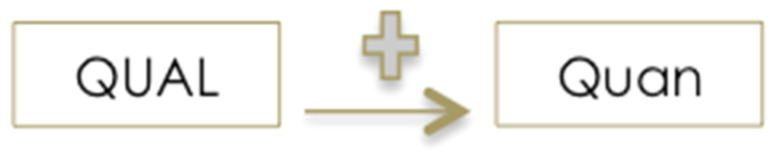
Methodologies description. Where the QUALITATIVE (QUAL) component predominates over the quantitative (quan) component, and special attention is paid to highlighting the development over time of the various stages: the results of each of these stages proved of interest for designing, fine-tuning, and interpreting the methodological design, so that the interrelationships among methodologies appear simultaneously (+) and sequentially (→) [60,62,87,88,89].

For integration of the results of the methodologies described above, we opted for a multimethod design of the type, see Figure 5.

**Figure 6:**
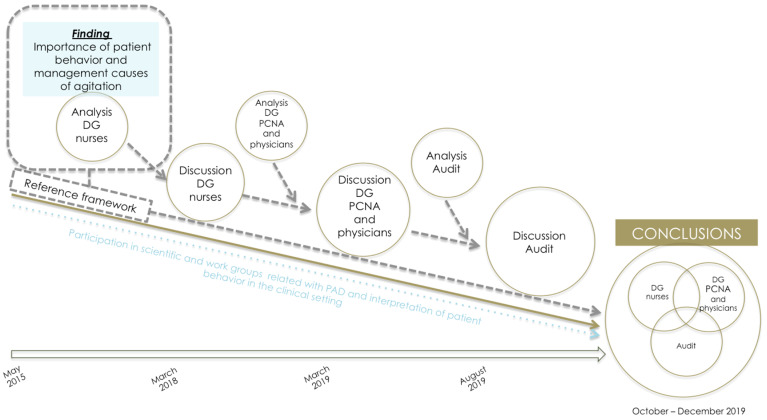
Cumulative discussion: integration of discussions of the different components via the “snowball effect”. Abbreviations: PCNA: patient care nursing assistants; DG: discussion group; PAD: pain/agitation-sedation/delirium.

As shown in Figure 6, the results of the DG held with the nurses drawn from ICU with FU were pooled with those yielded by DG with nurses drawn from the ICU with the OU of PR. Through this process of pooling the results of the DG involving nurses from both types of ICU, and a review of the literature addressing the interpretation of patient behavior and management of agitation, we constructed a theoretical reference framework with respect to PR use in critical patients. This reference framework was then used to design the script and materials used for the DG held with physicians, and served as a guideline for analysis of both the PCNA and physicians’ DGs and of the results of the audit.

The methodology used, albeit including stages of qualitative and quantitative research, was not conceived as a mixed design, in light of the absence of some of the elements considered crucial for a mixed approach such as dependence on the comprehension of the secondary design components. As explained, rather than adopting a mixed approach, we opted instead for the coexistence of different stages with different designs (a multimethod approach), geared to feeding into and enriching the subsequent stages to ultimately obtain a broad, in-depth, holistic, and pragmatic overview of what is regarded as a significantly complex research phenomenon [60,61,62,63,64,65,66,67,68,69,70,71,72,73,74,75].

Accordingly, the fullest integration of results took place in the discussion of those same results, where, thanks to “dynamic reflexivity”, a “snowball effect” was set in motion: knowledge generated in the preceding stages served to engender and build up a cumulative discussion with a broad scope (Figure 6).

## 4. Findings and Discussion

As a consequence of this cumulative and progressive integration of the findings of the different study components across the research process, a description of the results displays aspects that are at once explanatory and comparative. In light of the latter, we considered it opportune to merge the Results and Discussion sections by way of providing an internal dialogue between the contributions of the different components of the multimethod study, and a contrast with other external evidence.

### 4.1. Characteristics of the Clinical Context

In order to contextualize the findings on the decision-making process surrounding PR use as well as the factors that modulate this process, we propose to begin by setting out some of the results of the clinical audit that convey the reality of PR use at the 17 ICUs where the study was undertaken.

With respect to the characteristics of the ICU and in reference to the level of health care complexity, it should be noted that the mean nurse:patient ratio was 1:2 (range 1:1.25 to 1:3.62). Furthermore, it is likewise important to note that only 41.18% of the ICUs had a specific PR-management protocol, with specific training having been given at two of these units (Table 6 and Table 7).

A total of 1070 patients were observed, and of these, 194 had some type of PR, amounting to a median prevalence of PR use of the four observation periods of 19.11% (min: 0%–max: 44.44%)), with wide variability in both the overall group of patients and in those with ETT (Table 7). These data are within the range of PR use found in other studies. While papers from the United Kingdom and Scandinavian countries report a prevalence of close on 0%, the equivalent figures were 76% in Canada, 39% in the USA, 43% in Italy, just under 23% in Holland, and 15.6 to 43.9% in Spain [1,21,27,90,91,92]. Our data indicate a prevalence of 42.10% (min: 0%–max: 76.92%) in patients with ETT. Studies undertaken in France, Japan, Jordan, and Canada report prevalence as high as 76.9% of patients with ETT [5,25,27,28,47,86,93].

The most common PR location was on both upper extremities (85%). The most frequent adverse effects were edema in the area of application (39.65%), and increased agitation-disorientation-delirium (20.35%).

In accordance with the prevalence of PR use, ICUs were classified into compliers/non-compliers with the standard (prevalence of 15% or less). Of the 17 ICUs, five (29.41%) met the standard (Table 7).

### 4.2. Awareness and Definition of the Problem: Safety and Prevention of Risk in Relation to PR Use

**Figure 7:**
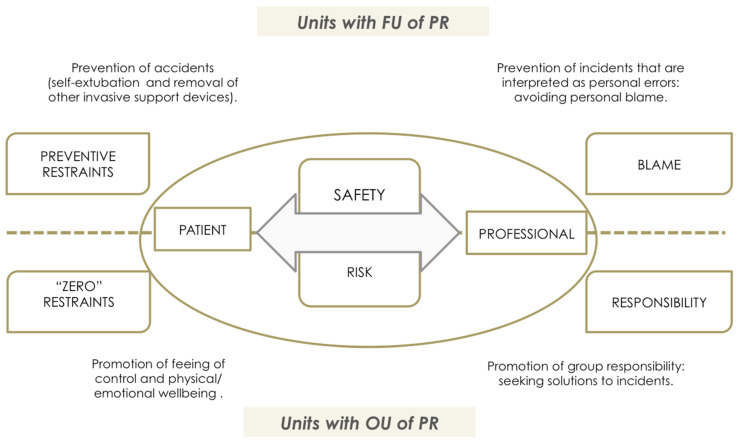
Concept of safety and risk in relation to use of physical restraints. Abbreviations: PR: physical restraint; OU: occasional/individualized use; FU: frequent/systematic use.

According to the participants’ discourse, the theoretical support underpinning PR use is the concept of safety and risk as perceived by health professionals. The concept of safety is thus envisaged from two perspectives that lie at opposite ends of a single continuum. These are safety in reference to the patient and safety in reference to the health professionals themselves vis-à-vis performance of their designated tasks (Figure 7).

At an ICU with a FU of PR, the PCNA’s discourse is based on the prevention of risk (essentially risk of self-removal of invasive devices), a standpoint from which they conceptualize this as “preventive restraint”, along with avoidance of “blame” for causing accidents.

“Preventive restraint” refers to routine and systematic (non-individualized) use of PR with the idea of ensuring a patient’s safety. This preventive attitude assumes that all patients admitted to ICU (mainly those from whom sedation is being withdrawn in order to advance toward removal of mechanical ventilation) are exposed to universal risk situations (not considering the particular situation to which the patient is exposed and how they interact with it). This risk of experiencing accidents (such as self-extubations) is attributed to patient behavior such as psychomotor agitation, without taking into account the causes or the magnitude of the consequences of the risk or of the preventive interventions (restraint) themselves.

DG mixed physicians: *“A patient who was awake was a little agitated and they already came in to put the wrist straps on him. I said: ‘‘What are you doing? I’m talking to him!’’ ‘‘No, no, it’s in case he becomes agitated”. Many patients, when they arrive back from operating theater, or if you’ve just intubated them, on come the wrist straps”*.

In relation with health professional-oriented safety, there is a culture focused on defensive intervention aimed at minimizing any possible incidents that might be attributed to the health professional. “Preventive restraint” thus becomes a defensive shield to prevent incidents whilst one is the person directly in charge of the patient. Similarly, there is a lack of communication within the team, and an absence of individualized assessment or consensus-based protocols.

DG2-Nurses: *“I feel that what you say about having the feeling of having your back covered is something that makes us afraid. It’s something that’s obvious, you probably lack the information and training at this level, and this lack of certainty of saying, “if certain things happen in what way could that affect me”, well it’s also something that leads you to say, “listen, first I protect myself, and..”*.

This would be in line with some of the results of the clinical audit, which bear out the fact that the PR-application profile fundamentally corresponds to patients with AAs (82.47%) and ETT (68.04%) (Table 8), with the most frequent indications for use being agitation (61.40%) and attempted self-removal of artificial airways (50.88%).

In contrast to “preventive restraint”, in units with OU of PR, the discourse of physicians, nurses, and PCNA is committed to the promotion of the patient’s well-being and comfort, rejecting protocolized interventions, and using individualized assessment to guide clinical judgment and decision-making. The goal here is PR use in the smallest number of cases, and always with an awareness (justified intervention, taking into account the possible complications arising from their use).

DG PCNA OU: *“[…] use restraints as little as possible […] and in full awareness […]. Well... I don’t get the idea of preventive (restraint). That’s to say, if we’re already saying that the fact of applying therapeutic immobilization is a complicated matter, if we apply it preventively...What do we do? Do we restrain everyone before waking them up?”*

DG mixed physicians: *“See what you’ve done? I told you that you had to have him restrained”, and that starts creating a dynamic […] it’s a question of the overall dynamic of people, that’s to say the feeling of thinking that one shouldn’t do it […] other than in specific cases and specific moments, it has to be a collective thing because if there’s somebody less inclined to do so, how do you put him on the right track with the idea, “See what you’ve done?”*

Furthermore, the concept of safety and professional risk is approached from a different perspective. Hence, at an ICU with OU, both physicians and PCNA see the risk of undesirable events as something intrinsic to professional practice, even though resources and advances in knowledge mean that these are less frequent and that safety in the workplace is far greater.

DG mixed physicians: *“When it comes to really inevitable risks [...] there are very few; what happens is that, with current state of knowledge, there are things that, as of now, we are unable to prevent, but if you analyze all the factors that influence the famous Emmental cheese with all those holes, if you start covering those holes, there’ll come a day when it’ll be very difficult for those errors and those mistakes to occur”*.

Thus, they do not perceive PR use as a means to prevent incidents that might reveal or highlight personal error in professional praxis: what they see instead is that the real solution to the occurrence of undesirable events is a feeling of group responsibility.

DG mixed physicians: *“…it’s the fear of feeling guilty, and that they might blame you for something which you think could be a personal error, when really it’s the same old story, we’re going to look for someone to blame and we’re not going to look for solutions and the reasons why things happen […] working together with the whole team and not trying to find scapegoats …but solutions”*.

DG-5-Nurses: *“Anyway, things have changed a little since safety protocols were established, like now everybody’s more aware that, instead of going around blaming people all the time, looking for someone to blame, it’s necessary to look for a solution to this type of problem. I feel that this whole concept is gradually changing”*.

In this context, the concept of safety acquires a more humanistic perspective, focused on the patient’s subjective experience and care from a holistic stance.

DG PCNA OU: [If we were to ask a patient, ‘What does being safe in an ICU mean to you?’, what would he say to us? Think about it]. *“Being cared for, […] being accompanied, not feeling isolated. The fact of knowing that he’s being taken care of, monitored, protected. That he’s in the hands of good health professionals […] that he has trust in the professionals”*.

With respect to this notion of health care quality and patient safety, in the clinical audit, an inverse correlation was found with overall PR prevalence and PR prevalence in patients with ETT of −43.15% and −52.14%, respectively (Table 9): in other words, those ICUs that registered better use of restraints in terms of quality displayed a lower prevalence of PR use, both in the overall group of patients and in the subgroup of patients with ETT.

In relation with the above, there is an evident need for a cultural change, a conclusion and at the same time, a demand, that other authors have also contemplated [2,8,38,94,95,96]. In this regard, the principal reason cited by health professionals to justify PR use (i.e., “preservation of patient safety related with life-sustaining devices”) should be modified and shifted toward other meanings of the term “patient safety”, based on an updated reference framework. Conceptualization of the terms “safety/risk” from a biologistic standpoint is predominant in health care models in which PR use is frequent [5,38].

### 4.3. Needs Assessment: The Clinical Reasoning Underlying the Decision-Making Process

The qualitative findings yielded by examining the experience of ICU nurses showed that in units with FU of PR, the presence of artificial airways or the initiation of the process of weaning patients off mechanical ventilation is used to justify the practice as a way of preventing self-removal of life-sustaining devices, without there being any assessment of the need for their use. Accordingly, PR use could not be attributed to a decision-making process for the management of restraints: a clinical judgment of this nature would not appear to exist.

DG mixed physicians: *“See what you’ve done? I told you that you had to have him restrained”, and that starts creating a dynamic that the patient is about to arrive, and the assistant says, “Do I restrain him?” What are you talking about, restrain him? He’s just come in, and you’ve got no idea of how he’s going to behave, he’s hardly in the room and you’re already restraining him. That’s why I say that it’s a question of the overall dynamic of people”.*

This would be in line with the results of the clinical audit, which concluded that in 33.68% of cases, PRs are applied as a matter of ICU policy (i.e., without reflexive evaluation of the need for PR use in each case) (Table 8).

While this reality might differ in an ICU with an OU of PR, where the health professionals acknowledge that such a decision-making process does indeed exist, they nonetheless experience difficulties when it comes to consciously putting it into words.

DG5 OU-nurses: *“…in each nurse’s judgment [...]. On what do you base yourselves in order to take the decision? […] if I see, well, that during these mornings the patient is calm, I take advantage of the situation, I unfasten him [...] quietly observing him to see how he reacts […]. I continue to keep an eye on him. [...] Don’t you use anything on which to base your decision, a RASS score, a delirium scale, some element that would enable you to decide? […] If he’s clearly agitated, we restrain him, then there’s no turning back […], in a calm patient who answers you by nodding his head, well I don’t know, you just keep on watching…”*.

In this connection, attention should be drawn to the differences found in the influence exerted by interpretation of the patient’s behavior on the indication for PR use or use of tools for assessment and management of the causes of agitation.

**Figure 8:**
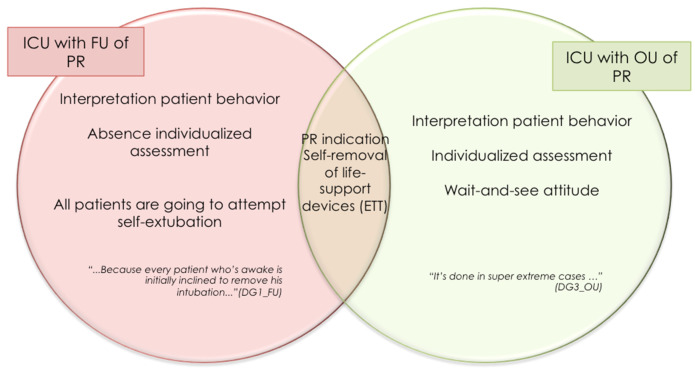
Venn diagram: influence of interpretation of patient behavior on the indication for use of physical restraints in intensive/critical care units with frequent versus occasional use of restraints. Abbreviations: PR: physical restraint; ETT: endotracheal tube; ICU: Intensive/critical care unit; OU: occasional/individualized use; FU: frequent/systematic use.

As can be seen in Figure 8, in both subtypes of ICU, the indication for the use of restraints is the prevention of the self-removal of life-sustaining devices, however, there are differences in the way in which nurses interpret patient behavior. At an ICU with FU of PR, it is assumed that all patients are going to attempt self-extubation. In contrast to this, an ICU with OU of PR adopts a wait-and-see attitude, with the decision to apply or not to apply restraints being taken in accordance with the assessment of each patient.

**Figure 9:**
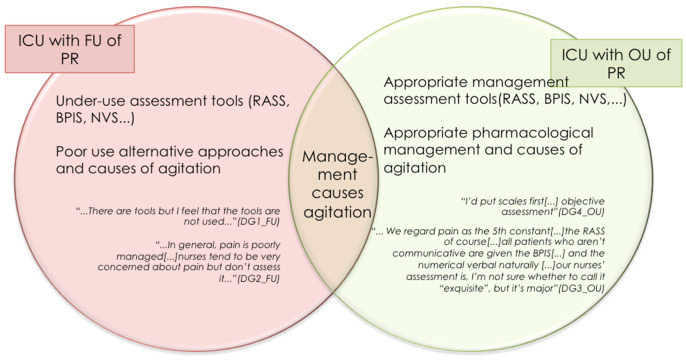
Venn diagram: under-use versus appropriate use of tools for assessment and management of the cause of agitation in units with frequent/systematic use of restraints versus units with occasional/individualized use of physical restraints. Abbreviations: BPIS: Behavioral Pain Indicator Scale; NVS: Numerical Verbal Scale; PR: physical restraint; RASS: Richmond Agitation Sedation Scale; ICU: intensive/critical care unit; OU: occasional/individualized use; FU: frequent/systematic use.

An ICU with OU of PR highlights the importance of the systematic use of validated tools for the assessment of pain, agitation/sedation, and delirium, with the use of these instruments being assumed to determine optimal use of pharmacological tools for the control of agitation. At an ICU with FU of PR, however, there is acknowledgment of the under-use of such tools and mismanagement of some causes of agitation such as pain (see Figure 9).

With respect to the above, the clinical audit found that, when it came to the monitoring of pain, 64.71% of ICU did so appropriately in communicative patients and 35.29% did so in noncommunicative patients. Whereas 88.24% of ICU monitored sedation in accordance with the guidelines, only 11.76% did so in the case of delirium.

We analyzed the relationship between compliance with the standard and variables of PAD monitoring as well as the use of protocols and training of health professionals (Table 9). Lower prevalence in the use of restraints was associated with appropriate monitoring of pain in noncommunicative patients (*p* < 0.001). With regard to the association with the monitoring of delirium, no conclusions can be drawn, given the negligible number of ICUs that monitored delirium appropriately.

In our results, the most frequent indications for use were agitation, attempted removal of AAs and other devices, which might be related with pain or delirium [2,29,97,98]. As with other authors, our data indicate that monitoring of pain in noncommunicative patients and delirium are the least reliable elements in the interpretation of patient behavior [21,94,99,100]. Even so, the association found between appropriate monitoring of pain in noncommunicative patients and low prevalence of PR use is noteworthy, a situation that could be accounted for by better interpretation of patient behavior: a better understanding of pain behaviors in noncommunicative patients (facial muscle tension, frequent movements, increased muscle tone, lack of adaptation to mechanical ventilation, etc.) brings one closer to a better diagnosis and its subsequent treatment, and takes one away from the generic diagnostic label “agitated patient”, under which PR are sometimes applied somewhat unthinkingly. This same result would have been expected in the case of the monitoring of delirium, but the low prevalence of appropriate delirium monitoring in the sample meant that this could not be analyzed. This idea seems to be reinforced by the results such as those published by Pun, Hsieh or Gu, who argued that appropriate detection of both pain (in communicative and noncommunicative patients alike) and delirium would reduce the profiles of agitation and need of PR [91,97,101].

### 4.4. Other Non-Clinical Elements That Influence Decision-Making about PR Use/Non-Use

In the participants’ discourse, it was possible to identify a large variety of elements that influence use/non-use of PR and respond to the conceptual framework of the “Iceberg Theory”, the theoretical reference framework adopted as the point of departure.

According to studies conducted in settings other than critical patients [3,16,17,37,39,43,48,50,102], health professionals cite the influence of individual, group, and organizational factors.

With respect to the first of these, mention should be made of: fear of physicians’ reaction to self-extubations *(“…if he should remove the tube, they’ll kill me…” [DG1-nurses]*); health professionals inflexibility when it comes to change *(“…because it’s always been done like that…”* [DG2]-nurses); and the interpretation put on patients’ behavior *(“...attempted suicides […] are restrained from the outset, this is a person who’s tried to commit suicide […] is not of sound mind […] no-one gives it a second thought [...] it’s automatic [...] it’s one of those cases in which…, well, it’s a textbook case…*” [DG1-nurses]).

Noteworthy among the group factors are: nurses being blamed for patient-related extubations (“…who’s to blame? The nurse […] your patient has become accidentally extubated... [...] It didn’t happen to the patient in the next bed, the nursing assistant or the doctor, but to you...” [DG1 nurses], “straight away they point the figure at you […] this was your mistake...” [DG2- nurses]); problems of communication between physicians and nurses (“...it depends on the nurse-doctor relationship...” [DG1-nurses], “...they don’t tell you that they’ve lowered the sedation dose...” [DG5-nurses]); pressure from the rest of the members of the group (“...You’ve also got to deal with your colleagues, which goes something like…And you’re not restraining him?’’...” [DG5-nurses]); and mismanagement in the unit of causes of agitation and assessment tools (“…we really do very little to find out what’s causing the agitation...” [DG2-nurses]), “....poor patient sedation ([…] he should be better sedated...” [DG5-nurses]). All of these are matters that could favor the use of restraints.

Finally, insofar as organizational factors are concerned, attention should be drawn to: restriction of family visiting hours (“…*the family only spends 20 min here during the morning...*” [DG1-nurses]); nurse:patient ratios; the architectural distribution of the ICU *(“…it should be an open-plan ward, because then anyone passes through and sees it..*.” [DG3- nurses]); and allocation of workloads *(“…we don’t follow them up because we don’t attend to them continuously...*” [DG1-nurses], *“... patient distribution, well you have one here and another there […] when it comes to sharing workloads, one of the things that should be borne in mind is patient safety*” [DG4-nurses]).

In connection with the organizational factors, though health professionals of both subtypes of units acknowledged the influence of the nurse:patient ratio, it was noteworthy that the ratios were similar in the two subtypes of ICU (i.e., OU and FU). Hence, although this factor is considered relevant by nursing professionals, it may possibly not be quite so decisive in clinical practice [26,39,45]. This hypothesis was explored using the results of the clinical audit by analyzing the possible relationship between optimal PR use, as shown by percentage compliance with the criteria considered, and institutional strategies (use of protocols or educational interventions), prevalence of PR, and nurse:patient ratios (Table 9). No statistical relationship was found with the existence of a protocol, specific training, or ratios. Along these lines, the literature indicates that there are a number of authors who initially singled out the ratio as a variable associated with increased application of PR. However, other studies have shown wide differences in the use of restraints in countries with the same ratios as the United Kingdom or northern European countries, which not only have an average nurse:patient ratio of 1:1, but which also include figures such as respiratory therapists in their staff rota [27,103]. Even so, we agree with Luk et al. and Kandeel and Attia in asserting that, despite the fact that an optimal nurse:patient ratio is more than desirable, the effect on PR prevalence attributed a priori to the ratio may not be all that decisive [23,103]. With regard to the profile of ICU staff, aside from nurse:patient ratios, a number of authors have highlighted the importance of ICU skill and training levels when it comes to decision-making about PR use [5,104,105].

### 4.5. Emotional Responses to Decision-Making about PR Use

In the discourse of health professionals drawn from ICU with both FU and OU of PR, the subtopic of “economy of feelings” surfaces, in which health professionals voice the absence of feelings generated by managing patients with PR.

DG1-nurses: *“...I don’t feel anything, good or bad...”*,

DG4-OU-nurses: *“...it’s part of your work, which you may or may not like to a certain extent [...] I don’t go home feeling any the worse…”*.

In line with the “economy of feelings”, related concepts emerge such as “nurse’s helplessness”, which appears as a stance with respect to problem-solving and decision-making so that nurses find themselves propelled toward the application of restraints when they perceive that they have been left without tools for managing the patient or have lost motivation and/or confidence in themselves and the resources at their disposal. In the same way, as postulated by the theory of learned helplessness (Seligman and Maier, 1967) [106], nurses, on seeing that their attempts to avoid PR use tend to fail systematically, allow themselves to be swayed by the dynamic of the unit *(“...applying restraints is also not the solution, that’s something we all understand, but what happens is that…” [DG3-nurses], “…it’s disagreeable, I also don’t like restraining them [...], but...”* [DG5-nurses]).

**Figure 10:**
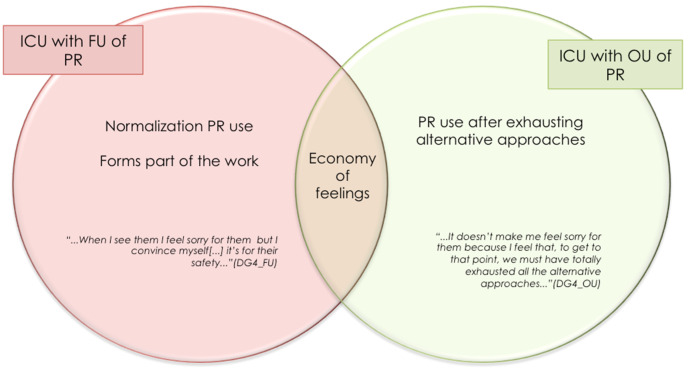
Venn diagram: economy of feelings with regard to the use of physical restraints in intensive/critical care units with frequent versus occasional use of restraints. Abbreviations: PR: physical restraint; ICU: intensive/critical care unit; FU: frequent/systematic use; OU: occasional/individualized use.

Although “economy of feelings” is envisaged in both subtypes of ICU, significant differences can be seen in the cause of this emotional blunting in the face of an extreme measure such as physical restraint: at an ICU with FU, normalization of PR use could account for the suppression/minimization of feelings; at an ICU with OU of PR, however, this blunting of feelings might be related to the fact that restraints are only used when all alternative approaches have failed (Figure 10).

While the “economy of feelings” referred to above might initially prove somewhat surprising, it is nevertheless in line with the data in the literature [16,43,107] on other spheres in which health professionals report an absence of feelings on considering the use of restraints as forming part of their work (normalization of PR use) [48]. Similarly, feelings of helplessness might justify the use of restraints as a nursing response in the face of the many problems that lie outside their professional scope [48,106,108]. Inappropriate management, the elements cited as modulators of PR use, and the simultaneous demand for safety, may place health professionals in a predicament that they seek to resolve by using restraints.

### 4.6. The Emergence of a New Paradigm for Guiding the Decision-Making Process: “Zero Restraints”

The idea of working under a “zero-restraints” paradigm appears naturally and credibly in the discourse between PCNA from ICU with OU of PR and physicians.

DG mixed physicians: *“When is it going to be of interest to us? Well, when everyone admits that what’s being done is not altogether right; perhaps we won’t get to 0% restraint use, but we can bring it down for sure […] there has to be some kind of aim or plan”*.

DG-5 Nurses: *“I believe we can restrain less because I’ve experienced it. Of course, the fact is I’ve seen it, so zero restraints, and I mean zero, zero, because I don’t know whether you’d need to restrain the patient in some specific case, but what I am sure of is that you can use restraints a lot less because truth is I’ve been there. And at the start, it was like “wow”. And when you see that you really could do it, when we made the break and continued with the same routine of making less use of restraints, well yes, there were people who removed the tube, but it wasn’t like we had a whole lot of extubations, more than before”*.

This approach is based on the following four premises: patient- and family-centered care; well-being-comfort and self-determination as outcome criteria; clinical judgment and planning of consensus-based interdisciplinary health care interventions; and the provision of adequate human and material resources (Table 10).

Regarding the patient as the subject of care implies taking patient safety as the axis of care, placing clinicians in the role of advocacy, with this being construed as a role of preserving the patients’ safety and defending their rights and interests [109]. From this standpoint, achievement of a subjective feeling of well-being and comfort is seen as a priority. Some key elements to facilitate such well-being are linked to the presence of a figure that would serve as a reference and effective support. In these participants’ discourse, this figure is represented by the nurse, though the family is seen as being able to contribute to the administration of emotional care with the appropriate support of nurses.

DG mixed physicians: *“And then there’s another factor […] all these non-pharmacological and non-healthcare measures, such as family accompaniment for instance, that’s to say, many patients are much calmer if they have a person, a relative at their side, who helps them when they come round, to focus and, to…, that’s been demonstrated, there are many non-pharmacological actions that depend on the family or relatives, who help a lot to maintain these situations”*.

DG-5 Nurses: *“I think that another measure you were talking about before to try and reduce the number of restraints would be to also increase visits, I feel that this would also be a possible measure. What we ourselves do in the case of some patients is to increase visits for family relatives. Patients are much better off with their family”*.

Furthermore, correct analgosedation is essential to place patients in the best condition to maintain their self-control and consciously participate in their recovery process. Providing the patient and family with information about clinical status and training in care are indispensable for making decisions and participating in caregiving, thereby achieving a feeling of control and self-esteem.

DG mixed physicians: *“[…] until a few year ago, it was thought that the intensive-care patient had to be sedated and not […] he has to be wide awake, collaborating and in no pain, and if you succeed in this, ok the intubation will cause a little discomfort, but well […]”*.

DG PCNA OU: *“[…] if instead of applying therapeutic immobilization […] and a member of the family came in and sat there […] holding the man’s hand, the fact is he was quiet the whole afternoon […] there was no need to restrain him or anything”*.

None of this is possible, however, without a consensus-based interdisciplinary approach based on in-depth knowledge of the patient and joint decision-making. A routine multicomponent assessment that envisaged PAD monitoring, the risk factors for its implementation, and the pertinence of the continuity of invasive devices, would be crucial in such a decision-making process. This integral and integrated interdisciplinary approach is reflected in the proposal of a “comprehensive restraints policy” and would imply the drawing up of joint-action protocols. In such a context, interpersonal communication (oral and written-recorded) is fundamental, as is formal regulation of the prescription of PR, which, being ambiguous, generates invisibility and a dilution of responsibilities.

DG PCNA OU: *“[…] we’re all colleagues. […] I’ve never had a problem with nurses when I’ve told them my opinion, but talking about it, you know? Not pejoratively, always ‘‘no, it’s just that the nurse’’, no, we also have our part”*.

DG mixed physicians: *“[…] just as a nurse will say ‘‘listen, we’ve got blood sugar levels rising to 250 mg/dL, open me a line of insulin’’, she should tell you, ‘‘listen, look, with this patient there’s no way of keeping him calm, he scares me’’, and we assess it together […] it’s about expressing a joint problem. […] we’re not at the bedside 24 h a day […] its left to the nursing staff, nurses are competent, and many things that you’re not going to see in the 10 min you spend there on your rounds every shift or hour […] it’s very curious how even nursing assistants. […]. Maybe it’s a little about bringing the key element […] cohesion […] into the team, most of the problems begin right there, in the lack of a common goal and communication”*.

With respect to human and material resources, different factors were identified that would foster the promotion of restraint-free ICUs (Table 10). Examples of these are: an adequate nurse:patient ratio that would allow for nursing proximity and bedside presence; pharmacological resources for elective analgosedation; and team training in aspects of assessment and psycho-socio-emotional interventions.

Finally, attention should be drawn to a key guiding factor cited in the health professionals’ discourses, namely, motivation. The motivation required here is the kind that would impel the team toward a change in organizational and health care culture, something that would imply, first, becoming fully aware of how one was working and what the aim of one’s work was, and second, planning a transition strategy endowed with resources that would favor a harmonious and integrating process based on communication, team work, shared leadership, and compassionate, empathetic, and committed care.

DG mixed physicians: *“Right, and no-one stops to think that maybe patients shouldn’t be restrained, and maybe we’re doing the wrong thing […] it’s also very difficult to break the… […] I see it as a problem that’s being trivialized and is something cultural, so it has to be changed [...] This has to be re-addressed as a non-trivial, non-routine measure, something that’s exceptional”*.

DG-3 Nurses: *“flexible positioning (is necessary). We’re dealing with persons and this isn’t a mathematical science: 2 + 2. There are many variables. Training and positioning (bearing in mind that everything’s relative, approximate and provisional)”*.

**Figure 11:**
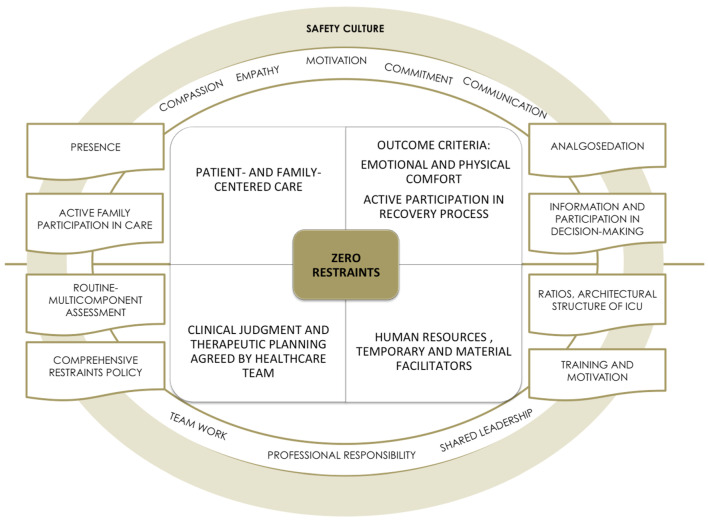
Work proposal under the “zero-restraints” paradigm. Abbreviations: ICU: intensive/critical care unit.

All these elements, highlighted as crucial to progress toward the “zero-restraints” work paradigm in ICU, are shown in Figure 11 below.

### 4.7. Joint Decision-Making: Overlapping Roles and Shared Responsibility in the Prescription of PR Use

Returning to the basic premises, by virtue of which patient safety would be construed as a collective responsibility, and decisions (the issuing of clinical opinions and treatment planning) would be based on consensus reached by health care teams, these give rise to the need to think about the prescription of PR use.

The findings show that physicians admit to trusting in the nurses’ judgment, considering them to have in-depth knowledge of the patients and their needs when it comes to guiding decision-making in the management of analgosedation and implementation of pharmacological measures (under nurse-driven protocols) as well as non-pharmacological measures. Furthermore, nurses are regarded by physicians and PCNA as the figure that centralizes information and serves as the manager of patient needs by coordinating therapeutic interventions. Furthermore, the nurse is acknowledged as being the professional carrying front-line responsibility for ensuring patient safety (with this being construed as a basic need or functional pattern). In this respect, their judgment, far from being questioned, is trusted and relied upon.

DG mixed physicians: *“If a nurse tells me that she’s going to apply restraints to a patient, even though I may not agree, I trust her judgment”*.

DG mixed physicians: *“It’s just that I have doubts about the world of prescription because, for example, restraints are considered something that should be prescribed by a doctor, it’s the sort of thing they say, but on the other hand, restraints, at least in my hospital, have always been regarded as coming under the heading of safety, let’s say, and safety is part and parcel of nursing, so there’s never been any challenge or argument about who had to prescribe, and it’s always been the nurses who’ve done it on their own account. They can report it, there isn’t any kind of secretiveness, it’s simply that they’ve viewed it as their initiative, no-one’s ever mentioned it…”*.

Notwithstanding the above, nurses demand support in decision-making, in that there are variables that influence and determine patient well-being and safety, and which depend on aspects of clinical and pharmacological management that come within the physicians’ scope of competence. The findings revealed opposing points of view, with feelings of empowerment on the one hand and vulnerability on the other.

DG Nurse-FU: *“In my unit, for instance, they’re never prescribed […] that’s surprising when physical restraints should actually be prescribed. And as I feel unsure, well you’re scared that one of these days something’s going to happen and you’re going have to face the music”*.

DG Nurse-FU: *“It seems to me that there‘s no reason why restraints should come within the sphere of doctors, but (prescription) is a way of protecting ourselves legally and drawing up protocols […] I feel that what’s lacking is training in the management of restraints, PR use protocols that are really for the safety of the patient”*.

What also emerges from the nurses’ discourse is the concept of “moral suffering” in relation to decision-making about the application of PR [5,41,110]. They refer to the fact that their use must be agreed upon and never imposed by physicians, alluding to the fact that this sometimes entails a moral dilemma for them (i.e., those who apply the measure but do not agree with the appropriateness of its prescription).

DG Nurse FU: *“But I’d like to explain to you that there are sure to be other measures to take and not just restraints, that a doctor can’t force you to restrain a person, because in that case, let him come down and do it himself. […] No, no, force me, no; force, no […] No, but maybe if he was the one who had to restrain the patient, then he might not be so ready to tell you to apply the restraints […] Yes obviously it’s really easy to delegate the job of applying a restraint or whatever, and that’s exactly why they don’t want to talk about it, because what they really do is delegate the job to somebody else, and that’s the person who actually applies the restraints”*.

**Figure 12:**
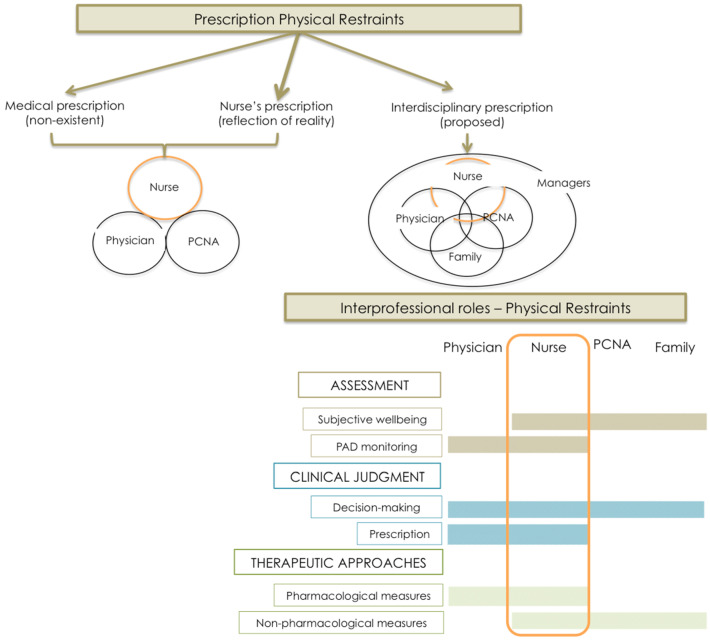
Proposed physical restraints prescribing and interprofessional roles. Abbreviations: PCNA: patient care nursing assistant; PAD: pain/agitation-sedation/delirium.

The current PR prescribing scenario at ICU around Spain seems to consist of the “nonexistence of medical prescription”, and the routinization and what one might call the “invisibilization” of decision-making [5,8]. In the participants’ discourse, however, the mere fact of airing and sharing points of view enables the possibility of joint prescribing to be raised as the ideal strategy for correct management of PR, acknowledging that it is a therapeutic measure that must be regarded as exceptional and, as a result, its prescription assessed with meticulousness, taking the views of the various members of the health care team into account. This proposal can be seen in Figure 12, which shows the distribution and overlapping of tasks and responsibilities in the inter-disciplinary approach to a common goal. This same approach is taken in Belgium where, even though the local nurses are legally entitled to prescribe PR, the Belgians provide that the decision should always be made jointly by the health care team as a matter of shared responsibility [111], something they describe as the “*interpersonal network as a forum for decision-making”*.

DG mixed physicians: *“[Prescription] really is something that we’ve left in the hands of the nursing staff and we pretty much try not to become involved […] Has it got to be a medical prescription? Well, I feel it ought to be a joint or delegated prescription. Seeing as it’s the nurse who’s at the bedside 24 h a day, or the nursing assistants. They should be the ones participating in this prescription, just as they participate in many other aspects of care. At present, it’s not like that, so in difficult situations they also look for support in the fact that there is or isn’t a medical prescription. Well, an agreement will have to be reached, it’ll have to be reviewed”*.

DG mixed physicians: *“This has to be re-addressed as a non-trivial, non-routine measure, something that’s exceptional, and that’s imbued with this exceptional nature because of its indications and joint prescription”*.

As has already been noted, on discussing the process of taking judicious and consensus-based decisions, anxiety, agitation, and rest-sleep feature prominently as fundamental pillars of pain assessment. Integrated multidimensional information leads to planning of health care interventions at a pharmacological and non-pharmacological level, which in turn implies impeccable coordination among the various professional figures. Having come thus far, it is necessary to introduce another actor with a say in matters and operational capacity, namely, the family, which may exert a major influence. Taking Haines’ contributions [112] as a reference, the family’s participation can be viewed as passing gradually from a transactional to a transitional phase and eventually becoming transformational, in other words, a progressive improvement in the dialogue between clinicians and the family. Hence, such a transformational participation would reach a maximum and translate as a true dialogue between clinicians and family as the result of a continuous interaction, which would guide decision-making, and ultimately lead to the design and implementation of pluralist decision-making models [113]. These models should evolve from the family-centered health care model toward a “family engagement” model (with the family empowered and actively included in decision-making from a stance of commitment), where the role of the family unit would become proactive and its needs would be met in line with its values [114].

In addition, the role of mid- and high-level managers should also be kept in mind. With regard to the latter, health care professionals highlight the passivity of managers with respect to PR use [5]. These results seem to be in line with the lack of involvement of managers (nurses as well as physicians and PCNA) detected in the discourse.

DG1-nurses: *“...the truth is, it’s always been done, everyone knows it, but no-one cares… It’s something that’s always there… and when I say no-one cares, I mean no-one… neither physicians, nor us, nor even my boss, who’s never given it a second thought”*.

DG2-nurses: *“...when there’s an extubation and they ask you if the patient was restrained… the physicians and the supervisor come to ask you why he wasn’t restrained…”*

Institutional positioning and involvement could be crucial in achieving changes in clinical practice, with the introduction of policies and strategic lines that would generate a culture of restraint-free care and provide the structural resources (material and human) and necessary knowledge to implement it [40].

## 5. Limitations and Strengths

This multimethod project was conducted throughout from a pragmatic and translational stance, and has sought to provide a broad overview of a complex reality. Indeed, acquiring in-depth knowledge of the reality surrounding PR and the factors associated with their use, opens up the possibility of reflexive management of restraints, which would in turn translate as a real reduction in clinical practice. In this respect, one of the undeniable strengths of the study is the use of multimethod strategies to minimize the specific limitations of each of the methodologies used. Even so, there are some limitations in each of the stages that warrant consideration.

### 5.1. Limitations Specific to the Qualitative Interpretative Phenomenological Component

In both stages of the qualitative interpretative phenomenological component, a comparative analysis was made of the discourse of health professionals drawn from ICU with OU or FU of PR. At the time, however, there were no real prevalence data on PR use at the ICU included. Hence, in light of the absence, both in the literature and at the ICU involved, of reliable data on the real prevalence of PR use and the modulating factors of such use, the ICU were stratified on the basis of a purpose-designed questionnaire, which though unvalidated, was nonetheless agreed upon by the whole research team.

### 5.2. Limitations Specific to the Audit Stage

Given the nature of the audit of the proposed clinical practice, a high number of cases were observed. Due, however, to the characteristics of this data-collection (no collection of individual data from each patient, thus conceivably including all the patients admitted, and omitting the need to sign the IC form), this inevitably gave rise to other related limitations such as the fact that the data were collected on an aggregate basis.

The set of optimal use criteria proposed is a model created by the authors, based on a review of the literature and knowledge of the study topic, but has not undergone validation, and must therefore be approached with caution and reviewed from a more up-to-date perspective, by, for instance, replacing concepts such as the fact that the patient is sedated by quantification with validated instruments (objective values of VAS/NVS or BPIS/BPS/CPOT).

### 5.3. Limitations Common to All Stages of the Study

The fact that only ICUs that voluntarily wished to do so participated in the study may influence the findings and exhibit an *excessively* benevolent image of PR use in this country.

The study included health professionals from a single region in Spain, and as a result, the conclusions must be contextualized and can only be extrapolated to cultural contexts with similar ethical conceptualizations such as other regions of this country or countries in the Mediterranean Basin.

The chronological order of the study and the time elapsed between the different stages may have amounted to a limitation in light of the changing nature of reality. Moreover, as observed in the Introduction, recent years have witnessed growing concern in ICUs about the management of critical patients’ behavior and, in a far more tentative manner, about PR use, with changes having taken place in recent years in the pharmacological and non-pharmacological strategies used for PAD management. Although the difference between theoretical advances and the translation of results to clinical practice is undeniable, with this being a long, and costly process, it is nonetheless likely that, across the years covered by this study, small changes may have occurred in the ICUs involved, with the result that, as of the date of writing, the reality recorded during the initial years may have undergone certain modifications. Finally, it is important to highlight the influence of the research process itself on the ICU/professionals involved, inasmuch as participation in the study may well have given pause for thought and, by extension, led to a change in these same units/professional participants.

## 6. Conclusions

The multimethod strategy has shown itself to be of great utility. Initially, the qualitative approach made it possible to gain greater insights into a complex and multifactorial phenomenon to then be able to validate some of the emerging hypotheses via the quantitative approach.

The various actors involved jointly build up a health care culture and a conceptualization of the terms “safety-risk”, which determine decision-making on the use of restraints at each of the intensive/critical care units.

The qualitative findings appear to confirm the “Iceberg Theory”: based on the discourses, a notion of risk and safety is identified that is constructed, transmitted, and interiorized in the context of a sociocultural group. These shared meanings are the germ of beliefs, values, and rituals which, in this case, determine the greater or lesser use of restraints.

This study revealed a reality of use that might be similar to that of other countries with frequent use of restraints.

Different profiles of PR use among the units studied were identified. The differences corresponded to aspects such as systematic use of tools for the assessment of pain/agitation-sedation/delirium, interpretation of patient behavior, the decision-making process, the significance attributed to patient safety and restraints, and the feelings generated by their use.

The restraint–free model requires an approach to safety from a holistic perspective, with the involvement of all team members and the family. Interventions such as interdisciplinary rounds, in which the nurse would act as the central reference figure, or the establishment of a model with quality standards for the application of restraints, may prove useful.

## Figures and Tables

**Table 1:** Specific research goals.

Qualitative Component		Quantitative Component
Stage I—Nurses	Stage II—PCNA and Physicians	Clinical Audit
To describe the nurses’ practical experience of decision-making in the placement, retention, and removal of PR. To identify the factors (individual, group, legislative) believed by ICU nurses to influence decision-making about PR use. To examine experience-based differences in PR use among nurses who work in ICU that make frequent/systematic, occasional/individualized, and mixed use of PR to ascertain how such experience influences the decision-making process surrounding PR use.	To describe the practical experience of PCNA and physicians with respect to the decision-making process (implementation, retention, and removal) relating to PR use in an ICU. To ascertain experience-based differences among PCNA and physicians according to their respective work settings in terms of PR use (frequent versus occasional), in order to determine the influence of such experiences on the decision-making process. To identify the factors that PCNA and physicians pinpoint as decisive for being able to work under the “zero-restraints” paradigm.	To analyze the relationship between PR use and pain/agitation-sedation/delirium (PAD) monitoring, nurse:patient ratios, and institutional involvement (existence of protocols and health professional training) in the application of these measures.

Abbreviations: PCNA: patient care nursing assistants; PR: physical restraint; ICU: intensive/critical care units; PAD: pain/agitation-sedation/delirium.

**Table 2:** Purpose-designed questionnaire for the stratification of intensive/critical care units by PR use.

Purpose-Designed Questionnaire for Stratification of ICU by PR Use
In what situations do PRs tend to be used in your unit? Are there some rules and regulations governing PR use? If so, what are they?; and to what situations do they relate? In your unit, is there some type of patient to whom PRs are systematically applied? In your unit is there some tool that acts as a guideline for the pertinence of PR use? How would you rate PR use in your unit, occasional or frequent?

Abbreviations: ICU: intensive/critical care units; PR: physical restraint.

**Table 6:** Characteristics of the ICU involved in the clinical audit stage.

General Characteristics of the ICU Involved	
Pain/agitation-sedation/delirium (PAD) monitoring	No. (%)
Appropriate monitoring of pain in communicative patients	11 (64.71)
Appropriate monitoring of pain in noncommunicative patients	6 (35.29)
Appropriate monitoring of sedation	15 (88.24)
Appropriate monitoring of delirium	2 (11.76)
Institutional variables	No. (%)
Specific PR protocol for ICU	7 (41.18)
Specific PR training in ICU	2 (11.76)
Nurse:patient ratio (mean)	1:2

Abbreviations: ICU: intensive/critical care unit; PR: physical restraint; no.: number.

**Table 7:** Characterization of intensive care units according to approximation to optimal physical restraint use, prevalence of physical restraints, prevalence of physical restraints in intubated patients, compliance with standard of prevalence of restraints, and nurse:patient ratios.

Characterization of ICU According to PR Use and Nurse: Patient Ratio									
	Opt. Use/ICU *	No. PR	No. pts.	Prev PR *	No. ETT	No. ETT and PR	Prev. ETT and PR *	Std. Compl.	Nrs.:pt. Ratio **
ICU 1	46.56% (32.5–47.99)	21	58	36.21%	19	11	57.89%	No	1:2.5
ICU 2	37.5% (35.94–39.37)	12	64	18.75%	19	8	42.10%	No	1:2.5
ICU 3	58.85% (52.60–60.16)	11	111	9.90%	50	5	10.00%	Yes	1:2.5
ICU 4	28.75% (28.75–28.75)	5	26	19.23%	12	5	41.67%	No	1:1.5
ICU 5	39.58% (32.81–44.27)	15	47	31.91%	16	11	68.75%	No	1:2.5
ICU 6	42% (38.43–46.07)	28	63	44.44%	26	20	76.92%	No	1:2.125
ICU 7	53.12% (43.75–62.5)	4	16	25.00%	6	3	50.00%	No	1:1.25
ICU 8	54.46% (53.12–57.81)	15	77	19.48%	32	12	37.50%	No	1:2
ICU 9	53.12% (52.60–57.03)	13	71	18.31%	26	9	34.61%	No	1:2
ICU 10	50% (46.87–56.25)	4	54	7.41%	13	2	15.38%	Yes	1:2
ICU 11	21.65% (20.78–25.67)	27	74	36.49%	33	20	60.61%	No	1:2.5
ICU 12	37.5% (31.25–45.75)	6	34	17.65%	6	3	50.00%	No	1:2.375
ICU 13	PR not used	0	85	0.00%	19	0	0.00%	Yes	1:2
ICU 14	43.75% (43.75–43.75)	1	101	0.90%	30	0	0.00%	Yes	1:2
ICU 15	31.25% (26.56–36.56)	13	68	19.12%	16	8	50.00%	No	1:2
ICU 16	24.85% (22.39–27.14)	17	66	25.76%	27	13	48.15%	No	1:3.625
ICU 17	59.37% (56.25–62.5)	2	55	3.60%	16	2	12.50%	Yes	1:2.5
	43.75% (29.46–53.12) Min = 21.65%–Max = 59.37%	194	1070	19.11% (9.90–25.75) Min = 0.00%–Max = 44.44%	366	132	42.10% (15.38–50.00) Min = 0.00%–Max = 76.92%		1:2

* Data expressed as medians (25th–75th percentile). ** Data expressed as means. Abbreviations: ICU: intensive/critical care units; PR: physical restraints; Opt. PR use: optimal physical-restraint use; no.: number; pts.: patients; prev.: prevalence; Compl. std.: compliance with standard (prevalence of physical restraints ≤ 15%); Nrs.: pt. ratio: nurse: patient ratio.

**Table 8:** General characteristics of patients WITH physical restraints observed in the clinical audit stage.

Characteristics of Patients with PR	
Prevalence of physical restraints	No. (%)
Prevalence of patients with PR	194 (19.11)
Characterization of patients with PR, according to AA and MV use	No. (%)
Patients with PR and AAs	160 (82.47)
PR and ETT	132 (68.04)
PR and tracheostomy cannula	28 (14.43)
Prevalence patients with PR and NIMV	7 (3.61)
Prevalence patients with PR, without AAs or NIMV	27 (13.92)
Indication for PR use	No. (%)
ICU policy	65 (33.68)
Agitation	119 (61.40)
Hyperactive delirium	69 (35.44)
Attempted self-removal of artificial airway	99 (50.88)
Attempted self-removal of other devices	88 (45.61)
Risk of falls	16 (8.42)
Observed behavioral changes	No. (%)
Total behavioral changes	64 (32.97)
Increase in agitation, disorientation, delirium	39 (20.35)
Decrease in agitation, disorientation, delirium	18 (9.12)
Crying, sobbing	3 (1.40)
Verbalization of feelings of humiliation, shame	4 (2.10)

Abbreviations: PR: physical restraint; no.: number; AAs: artificial airways; VM: mechanical ventilation; ETT: endotracheal tube; NIMV: non-invasive mechanical ventilation; UE: upper extremities; LE: lower extremities; ICU: intensive/critical care unit.

**Table 9:** Variables associated with optimal use of physical restraints.

Variables Associated with Optimal PR Use				
		n	Optimal PR Use (Median) *	*p*-Value
Specific PR protocol for ICU				0.3165
	YES	6	46.87%	
	No	10	39.75%	
Specific PR training in ICU				0.8238
	YES	1	43.75%	
	No	15	42.01%	
Nurse:patient ratio (median) **		--	−23.47%	0.3820
PR prevalence **		--	−43.15%	**<0.001**
PR prevalence in patients with ETT **		--	−52.14%	**<0.001**

* Over total (n) sample of 16 ICUs (degree of approximation to optimal use not ascertainable in one ICU due to PR not being applied throughout the observation period). ** Spearman correlation analysis. Abbreviations: PR: physical restraint; ICU: intensive/critical care unit; ETT: endotracheal tube. The use of bold is to highlight the results in which statistically significant differences were found.

**Table 10:** Factors favoring PR-free intensive/critical care units.

Factors Favoring PR-Free ICU	
Empathetic attitude	“It’s extremely negative to come round from anesthesia or sedation, not knowing where you are, you’re disoriented, you don’t know anybody, and what’s more, you’re about to move and you can’t.” DG PCNA OU
Compassionate treatment relationships	“But it’s also because it’s something we don’t care about, it’s that we don’t get involved, it’s that it’s better to be, say, seated at the computer, and I say it myself, the dehumanization that’s going around now is just that, we’re there sitting at the computer, on the Internet, rather than being with the patient. And I’ve seen many patients, no lie, when they’re intubated, they become very agitated, also because we don’t try to make an effort to understand them; you can understand a patient in a case where he’s intubated and no other measures are being found for him, but it’s that we pay no attention, really, you know? The fact is, it’s very sad.” DG PCNA FU
Team work	“Information. Information, communication, between the healthcare workers, between the members of the team.” DG PCNA FU
Human resources adapted to the unit’s needs	“It’s just that all this would have to be analyzed and, OK, maybe it’s impossible for them to give you more people, but, perhaps it would be possible to divide up…well look, since this guy is... -you attend to this one and I’ll see to those- you know what I mean?.” DG PCNA OU
Unit structure that would allow for patient monitoring and easy access	“Well, in my unit, the good thing we have is that it’s open plan and there are no walls. So I’m watching my patient continuously. That’s very good because it allows you to monitor your patient remotely, and it also calms and reassures him to know you’re watching him, even though you may have to make a run for it.” PCNA FU
Training of healthcare staff	“And that was how it was until recently when the course was held and things changed a bit.” DG PCNA OU
Information from the patient and family	“But the thing is that one has to start by explaining to him not to get upset. Other ways of talking to him, I feel that...” DG PCNA OU
Participatory presence of the family	“Yes, but with immobilization […] if instead of applying therapeutic immobilization […] the other day it happened to us, instead of doing that, a member of the family came in and sat there holding the man’s hand, and the fact is he was quiet the whole afternoon. I mean to say, it was as if he wasn’t there. I took his temperature, he had lunch, he had dinner, we put him in the armchair, and there was no need to restrain him or anything.” DG PCNA OU

Abbreviations: ICU: intensive/critical care unit; PR: physical restraint; DG: discussion group; PCNA: patient care nursing assistants; OU: occasional/individualized use; FU: frequent/systematic use.

## Data Availability

Data are stored under the custody of the main researcher.
